# Combination of Antibodies and Antibiotics as a Promising Strategy Against Multidrug-Resistant Pathogens of the Respiratory Tract

**DOI:** 10.3389/fimmu.2018.02700

**Published:** 2018-11-20

**Authors:** Mirian Domenech, Julio Sempere, Sara de Miguel, Jose Yuste

**Affiliations:** ^1^Centro Nacional de Microbiología, Instituto de Salud Carlos III, Madrid, Spain; ^2^Centro de Investigación Biomédica en Red de Enfermedades Respiratorias, Madrid, Spain

**Keywords:** antibodies, antibiotics, resistance, respiratory infections, immune response

## Abstract

The emergence of clinical isolates associated to multidrug resistance is a serious threat worldwide in terms of public health since complicates the success of the antibiotic treatment and the resolution of the infectious process. This is of great concern in pathogens affecting the lower respiratory tract as these infections are one of the major causes of mortality in children and adults. In most cases where the respiratory pathogen is associated to multidrug-resistance, antimicrobial concentrations both in serum and at the site of infection may be insufficient and the resolution of the infection depends on the interaction of the invading pathogen with the host immune response. The outcome of these infections largely depends on the susceptibility of the pathogen to the antibiotic treatment, although the humoral and cellular immune responses also play an important role in this process. Hence, prophylactic measures or even immunotherapy are alternatives against these multi-resistant pathogens. In this sense, specific antibodies and antibiotics may act concomitantly against the respiratory pathogen. Alteration of cell surface structures by antimicrobial drugs even at sub-inhibitory concentrations might result in greater exposure of microbial ligands that are normally hidden or hardly exposed. This alteration of the bacterial envelope may stimulate opsonization by natural and/or specific antibodies or even by host defense components, increasing the recognition of the microbial pathogen by circulating phagocytes. In this review we will explain the most relevant studies, where vaccination or the use of monoclonal antibodies in combination with antimicrobial treatment has demonstrated to be an alternative strategy to overcome the impact of multidrug resistance in respiratory pathogens.

## Introduction

One third of the annual deaths occurring in the world are estimated to be due to infectious diseases and notably, infections affecting the respiratory tract are responsible of 4 million deaths worldwide (1). According to estimates of the World Health Organization, pneumonia kills more children worldwide than any other disease, even more than acquired immune deficiency syndrome (AIDS), malaria and measles combined (2–4). In adults, the impact of community acquired pneumonia (CAP) or nosocomial pneumonia (including hospital-acquired pneumonia and ventilator-acquired pneumonia) is also very worrisome as they are associated with remarkably high morbidity and mortality rates worldwide (5). One the major causes of these pathologies is *Streptococcus pneumoniae* (pneumococcus) that has greater incidence in children under 5 years old and adults over 60 years old, although the mortality is much higher in elderly population worldwide (4, 5). Pneumococcus is indeed, the main etiologic agent of CAP, as well as, non-epidemic bacterial meningitis and acute otitis media (AOM), but is also one of the major causes of bacterial sepsis (6). Other frequent causes of CAP include *Haemophilus influenzae, Pseudomonas aeruginosa, Staphylococcus aureus*, and also other pathogens grouped as atypical bacteria (including *Mycoplasma* spp, *Chlamydia* spp, and *Legionella* spp) (7).

The search of effective treatments to fight against infectious diseases has been, since many years, among the main challenges of medicine. Before the discovery of antibiotics, there were very few choices against bacterial infections. In the last decade of XX century, therapies based in antibodies to treat these infections were commonly used (8) and, in the 20's of last century, serum therapy was used against many bacterial diseases including infections affecting the respiratory tract, such as those caused by *S. pneumoniae* (9). These treatments reduced in a 50%, the mortality caused by this pathogen (10). However, when antibiotic chemotherapy emerged in the 30's decade of last century, serum therapy was abandoned and it was substituted by antibiotic treatment due to its higher effectivity and lower toxicity. Interestingly, the appearance of resistant strains appeared promptly after the general use of antibiotics. Resistance to several antibiotics is a common phenotype in the majority of these pathogens including multidrug resistant (MDR) strains of pneumococcus. In some cases, such as extended spectrum β-lactamase producing enterobacteriaceae and methicillin-resistant *S. aureus* (MRSA) dissemination of resistance has become a serious threat worldwide (7). In the last years, the use of monoclonal antibodies has been proposed as an alternative for the treatment of MDR pathogens, due to their marked specificity against the bacterial pathogen, their limited possibility of creating resistance and their ability to act synergistically with antibiotics (11). A different approach to reduce the burden of disease caused by MDR pathogens and also limit the dissemination of resistance genes is based in the implementation of effective vaccines with high coverage rates among the pediatric and adult population (12, 13).

## Impact of vaccination against antibiotic resistance in respiratory pathogens

To control antibiotic resistance, vaccines have been proposed as promising intervention measures to control the spread and dissemination of MDR strains. Indeed, existing vaccines against important pathogens, such as *S. pneumoniae* or *H. influenzae* type b may contribute to reduce the burden of antimicrobial resistance (14–17). One of the best examples is the reduction of MDR serotypes after the introduction of pneumococcal conjugate vaccines. Hence, preventive and therapeutic measures against infection produced by *S. pneumoniae* have modified the resistance pattern of this pathogen. PCV7 and later PCV10 and PCV13, are pneumococcal vaccines containing the capsular polysaccharides of the main serotypes causing invasive pneumococcal disease (IPD) protecting against the most common serotypes that are resistant to antibiotics. These vaccines were commercialized at the beginning of this century to promote immunization against pediatric population, although PCV13 is also indicated for adults. The general use of these vaccines induced a drastic decrease of the incidence of IPD caused by serotypes included in the vaccines and also reduced the prevalence of non-susceptible serotypes to antibiotics (18–20). As a consequence, PCV7 and later PCV13 have had a clear impact in the epidemiology of clinical isolates obtained from adults, who have been indirectly beneficiated from pediatric vaccination (18–22). Another example is vaccination against *H. influenzae* type b that has reduced the overall morbidity and mortality by this microorganism showing an impact on antibiotic resistance by declining ampicillin resistant strains (23). Additional evidence is the influenza virus vaccine and how can diminish the impact of antibiotic resistance in bacterial pathogens affecting the respiratory tract. Although, the best studied interaction of influenza virus with a bacterial specimen is with *S. pneumoniae* (24–26), there are many studies demonstrating possible associations between influenza and other respiratory bacterial pathogens, such as *S. aureus, H. influenzae, Streptococcus pyogenes*, and *Neisseria meningitidis* (27–29). Preventing infection by influenza virus due to vaccine strategies, may decrease the subsequent infection by some of the bacterial pathogens mentioned above, which in some cases may harbor high levels of antibiotic resistance.

## Immunomodulatory effects of antibiotics

The emergence of strains with high levels of antibiotic resistance might jeopardize the success of the antibiotic therapy (30). Antibiotics play their role against bacteria in a more complex mechanism when they exert its activity *in vivo* in comparison to the *in vitro* conditions due to the presence of serum proteins and components of the host immune response (31). Immunomodulation mediated by antimicrobial drugs can be explained as an induction of immunity to pathogens triggered by the chemotherapy compound. In this sense, immunoglobulins and complement components can improve the activity of β-lactam antibiotics (32, 33) whereas the presence of albumin and globulins limit free-drug plasma concentrations affecting the expected antibacterial *in vitro* effect (34–38). This effect is only relevant if the binding to plasma proteins is high (more common in cephalosporins than in penicillins) (37, 38). However, other authors using a pharmacodynamic simulation at physiological conditions including binding proteins levels similar to those found in humans, demonstrated that the presence of binding proteins did not impair the anti-pneumococcal activity of cefditoren (CDN), which is a high binding protein cephalosporin (39). β-lactam antibiotics display its antibacterial activity by a direct action against the microorganism. However, IPD is associated to high levels of morbidity and mortality despite an appropriate antibiotic therapy (40). The lack of antibiotic efficacy is very common being especially evident in immunocompromised patients, suggesting that the recovery of these patients depends on the joint action of antibiotics and the host defense mechanisms.

Alteration of bacterial surface structures caused by certain antibiotics might contribute to a major exposition of antigenic epitopes that are deeply or hardly exposed. This greater exposure might promote the opsonization by different components of the host immune response, such as acute phase proteins, enhancing the recognition of the respiratory pathogens by professional phagocytes. Pentraxins, such as C-reactive protein (CRP) and serum amyloid P component (SAP) are the main acute phase proteins in human and mice, respectively (41). CRP levels increase during different respiratory infections, demonstrating the importance of this protein as a sentinel molecule (42). One of the most important functions by CRP and SAP in host defense against invading pathogens is the opsonization of microorganisms and later the activation of the phagocytosis process by Fcγ receptors (41, 43–45). In this sense, it has been demonstrated that the recognition by CRP and SAP of different clinical isolates of *S. pneumoniae* is enhanced when the bacteria is opsonized with serum containing sub-inhibitory concentrations of β-lactams, suggesting that these antibiotics allow these pentraxins to recognize *S. pneumoniae* in a more efficient manner increasing the phagocytosis (32, 33). A different acute phase protein termed pentraxin 3 also has demonstrated to be very effective in combination with antimycotic drugs against infections produced by *Aspergillus fumigatus*, stimulating the antifungal activity of phagocytes (46). Moreover, the use of cephalosporins has been associated to an increased in the serum bactericidal activity against important pathogens, such as *Escherichia coli* and *P. aeruginosa* (47, 48), whereas the treatment with erythromycin (ERY) seems to produce small rupture points (breakpoints) in the cell wall, causing the breakage of the envelope of *Legionella pneumophila* (49, 50). In addition, it has been demonstrated that the macrolide azithromycin (AZM), in concentrations lower than the minimum inhibitory concentration (MIC), destabilizes the outer membrane increasing the permeability and producing the death of *P. aeruginosa* (51).

As an alternative, antibiotics might reduce the expression of certain virulence factors involved in the inhibition of complement activation and phagocytosis. Indeed, a recent study has confirmed that certain antibiotics in sub-inhibitory concentrations modify the expression of virulent genes of *S. aureus* (52). An additional explanation for the enhanced activation of the host immune response by macrolides could be related to its mechanism of action as these antibiotics interact with the ribosomal 50s subunit inhibiting the protein biosynthesis (31). This is an important aspect in terms of pathogenesis as sub-inhibitory concentrations of macrolides inhibit the production of pneumolysin (Ply) which is an important virulence factor involved in C3 evasion (53, 54). Furthermore, certain macrolides, such as ERY, AZM, or clarithromycin, inhibit negatively the synthesis of Ply and pneumococcal surface protein A (PspA) (55–57). This is relevant from the antimicrobial and immunological perspectives because the combination of both proteins has an additive effect and is very effective inhibiting the activation of complement immunity (54). Additional evidence demonstrate that macrolides exhibit immunomodulatory effects by inhibiting neutrophil inflammation and macrophage activation, reducing the levels of Th2 cytokines which might be important for the treatment of chronic inflammatory diseases using this antibiotic (58).

Overall, antimicrobial drugs can trigger the humoral and cellular response using a broader range of mechanisms including the recognition by acute phase proteins and complement-mediated immunity, inhibition of bacterial virulence factors involved in immune evasion and reduction of the inflammatory response.

## The host immune response against respiratory pathogens is boosted by the cooperation of antibiotics and antibodies

One of the major risks of respiratory infections is that are frequently associated to high morbidity and mortality rates despite appropriate antibiotic therapy with poor prognosis when the infective pathogen is highly resistant to the antibiotic prescribed (40). In the absence of antibiotic treatment, the outcome of the infection depends on the balance of the interaction between bacterial virulence factors and host defense mechanisms. Antibiotics normally display their antibacterial activity by a direct action against the microorganism. Clearance of respiratory pathogens from the systemic circulation depends on the opsonization by the complement system and the phagocytosis process (59, 60). In this sense, it has been observed that antibodies bound to *Cryptococcus neoformans* modify the genetic expression and the metabolism of certain genes, increasing the susceptibility of the pathogen to different antifungal drugs (61). Vaccination against respiratory pathogens including *S. pneumoniae*, induces the generation of specific antibodies that can interfere with the growth and metabolism of different microorganisms, suggesting a novel mechanism for antibodies mediated immunity (62). The damage produced on the surface of the pathogen by antimicrobial drugs might allow certain components of the cellular envelope to be more accessible and therefore, improve the recognition by complement components and specific antibodies. For example, exposure of MDR strains of *Klebsiella pneumoniae* to serum and β-lactam antibiotics increased the C3 levels on the bacterial surface (63).

**Figure 1:**
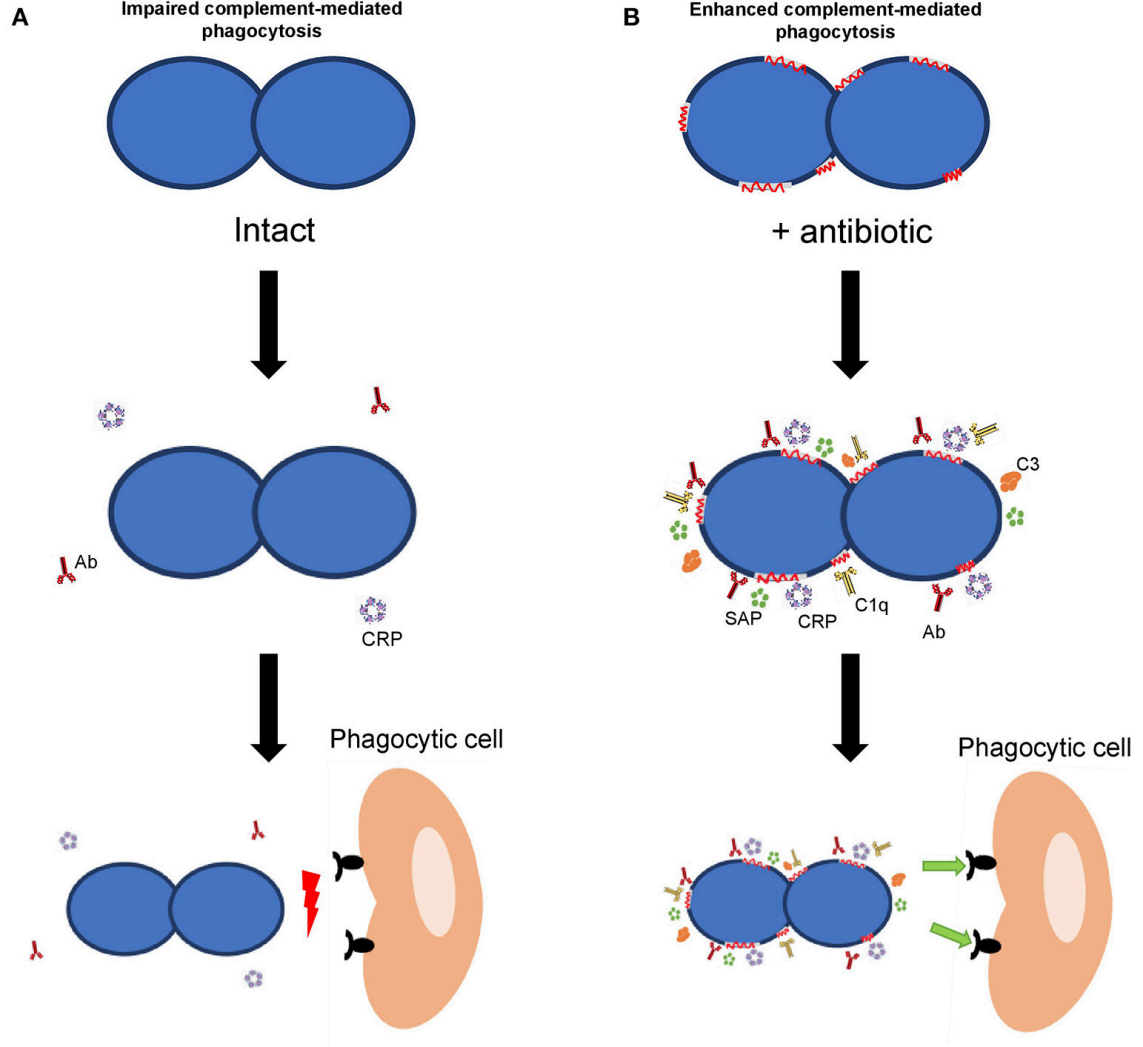
Effect of host immune components on bacterial opsonization and phagocytosis. **(A)** Interaction of the host immune response with a bacterial pathogen in the absence of antibiotics. **(B)** Interaction of different components of the host immune response in the presence of antibiotics. In the presence of antibodies, acute phase proteins (CRP and SAP) and complement components (C1q and C3), the recognition of the bacterial pathogen is greatly enhanced when antibiotics are present (right panel).

Considering *S. pneumoniae*, the classical pathway of the complement system is the most important pathway for complement activation (64, 65). Activation of this pathway *in S. pneumoniae* was significantly increased in the presence of β-lactam antibiotics confirming that alterations caused by these antibiotics, even at sub-inhibitory concentrations, improve the complement mediated immunity by a mechanism that is dependent on the activation of the classical pathway (33). Once the complement cascade is activated and after numerous enzymatic reactions, the key component C3 is formed. In the presence of serum containing antibodies to pneumococcus and sub-inhibitory concentrations of β-lactam antibiotics or macrolides, C3 deposition on the surface of different MDR strains was markedly increased (33, 66). Overall, activation of the recognition by acute phase proteins and complement proteins by certain antibiotics, such as β-lactams and macrolides in the presence of specific antibodies has functional consequences increasing the phagocytosis process and the clearance of the microorganism (Figure 1) (32, 33, 66). Previous studies using a sepsis model of infection in mice, have demonstrated that protective doses of amoxicillin and cefotaxime were, approximately, eight times lower in the presence of antibodies than in their absence (67, 68). It is important to mention that whatsoever, the possible benefit of the synergistic effect mediated by antibodies will not be based in a reduction of the antibiotic doses. The benefit would be to obtain a higher efficacy from the therapeutic perspective after the administration of the common doses used against IPD cases produced by clinical isolates with high MIC levels (32, 33, 66, 67). Hence, vaccination reduces the magnitude of the pharmacodynamics indices (i.e., drug exposure defined from pharmacokinetic/pharmacodynamic ratio, ft > MIC, fCmax/MIC or fAUC/MIC), needed to reach a certain effect. Consequently, in the presence of antibodies, usual doses of the antibacterial agent would necessarily cover clinical isolates with higher levels of resistance (32, 67).

The early onset of antibiotic treatment is essential to prevent the spread of the bacterial pathogens through the respiratory tract and the dissemination to the systemic circulation because any delay initiating the treatment may lead to the high fatality rates associated to respiratory infections (69). This problem gets worse when the bacterial pathogens harbor high levels of antibiotic resistance. In this case, treatment with β-lactams or macrolides may suppose a new strategy to reduce the possibility of treatment failure in those individuals who have been previously vaccinated against *S. pneumoniae*. This assertion is based in the enhanced efficiency of the host immune response to clear the bacterial pathogen in the presence of specific antibodies and these antibiotics (32, 33, 66–68, 70). This cooperative effect between antibodies and antibiotics it seems to be limited to β-lactams and macrolides because the presence of sub-inhibitory concentrations of levofloxacin and specific antibodies did not affect the opsonization by C3 against *S. pneumoniae* (66). These results explain why the treatment with sub-inhibitory concentrations of levofloxacin in mice previously immunized against *S. pneumoniae* did not increase the survival rate (71). Boosting effects on the host immune response by macrolides have been studied in other bacterial pathogens including Gram-positive and Gram-negative bacteria (31). Antimicrobial activity of macrolides is increased against resistant strains of *E. coli* and *S. aureus* when clinical isolates are exposed to sub-inhibitory concentrations of ERY and AZM in the presence of human serum. In the case of *S. pneumoniae*, exposure of resistant strains to sub-inhibitory concentrations of different macrolides increased C3 activation on the bacterial surface (66). Moreover, in the absence of the main autolytic pneumococcal enzyme, the amidase LytA, C3 deposition remained altered regardless the presence of opsonic antibodies and antibiotics demonstrating that LytA play a key role in the recognition by the complement system (66).

The rise of drug resistance to the majority of all antibiotic classes is particularly critical from the therapeutic perspective within the designated ESKAPE pathogens (*Enterococcus faecium, S. aureus, K. pneumoniae, A. baumannii, P. aeruginosa*, and *Enterobact*er spp.) (72). To fight these infections, the use of monoclonal or polyclonal antibodies has been proposed as antimicrobial alternatives against MDR strains including ESKAPE pathogens, with several antibodies being tested in different phase I-IV clinical trials (11, 73, 74). The possibility that these antibodies might confer boosting effects with antibiotics is a promising field to explore. In this sense, polyclonal antibodies to efflux pump proteins of *Stenotrophomonas maltophilia* have demonstrated additive or synergistic effects with a variety of antibiotics including cotrimoxazole, ticarcillin–clavulanate, and ciprofloxacin (75).

In *P. aeruginosa*, bispecific antibodies targeting the serotype-independent type III secretion system (injectisome) virulence factor PcrV and persistence factor Psl exopolysaccharide have shown to be very effective increasing the antimicrobial activity of different antibiotics against MDR strains (76, 77). Synergistic activity of these antibodies with ciprofloxacin, meropenem, ceftazidime, and tobramycin was observed, demonstrating enhanced effect against lung injury and prevented bacterial dissemination from the lung to the systemic circulation (76, 77). In addition, the use of panobacumab which is an IgM/κ monoclonal antibody directed against the LPS O-polysaccharide moiety of *P. aeruginosa* in combination with meropenem significantly increased bacterial clearance in the lung confirming the benefits of the joint therapy against MDR strains of this important pathogen (78).

In the case of *S. aureus*, monoclonal antibodies targeting different toxins and virulence factors have demonstrated synergistic effects in combination with several antibiotics. Therapeutic administration of a monoclonal antibody against the pore-forming toxin, alpha toxin in combination with vancomycin or linezolid resulted in improved survival against induced pneumonia by reducing inflammation and lung damage (79, 80). This combination results in a more robust immune response leading to reduced disease severity and accelerated healing relative to those with linezolid or vancomycin monotherapy. As a consequence, addition of antibodies to alpha toxin to antibiotic monotherapy may provide a benefit over antibiotics alone through its complementary mechanism of action (79, 80). Similar results were observed by other authors using monoclonal antibodies against different staphylococcal cytotoxins including alpha hemolysin and leukocidins demonstrating synergistic effects in the combination with linezolid that allowed a significant increment in survival rates (81). Among the numerous staphylococcal toxins, enterotoxin B has been classified as a class B biological warfare agent. Monoclonal antibodies to this toxin in combination with vancomycin increased survival rates and altered cytokine responses, compared with monotherapy with either monoclonal antibody or vancomycin alone (82).

Another warfare pathogen for which joint therapy using antibodies and antibiotics has been proposed is *Bacillus anthracis*. The most lethal route of exposure is via inhalation, and the disease is characterized by extensive bacteremia and toxemia which, without aggressive prophylaxis or intervention, results in a high mortality rate mainly due to anthrax exotoxin-driven pathogenesis. Monoclonal antibodies to the anthrax toxin protective antigen in combination with levofloxacin or doxycycline resulted in increased survival compared to the antibiotic alone and would provide an effective therapeutic strategy against symptomatic anthrax, even late in the course of the disease (83, 84).

Finally, a randomized, double-blind, placebo-controlled study of two neutralizing, fully human monoclonal antibodies against *Clostridium difficile* toxins A (CDA1) and B (CDB1) demonstrated that the addition of these antibodies to antibiotics metronidazole or vancomycin, significantly reduced the recurrence of *C. difficile* infection (85).

## Concluding remarks

The use of prophylactic measures including vaccination or even the use of monoclonal antibodies to treat or prevent severe infections caused by MDR pathogens is a realistic approach to fight these infections and reduce the impact of antimicrobial resistance in respiratory pathogens. The ability of certain antibiotics of showing an immunomodulatory effect which is strongly enhanced by the action of the host immune response is an alternative and promising strategy to eradicate or at least ameliorate the impact of MDR bacterial isolates in clinical practice. Further research in this field will contribute to identify and characterize novel prophylactic and therapeutic measures that in combination with current antimicrobial drugs may be effective solutions against the emergence of MDR pathogens, limiting their impact in public health.

## Author contributions

MD, JS, SdM, and JY prepared the text. JS and JY produced the included figure. All authors assisted in the conception of this review, interpretation of the relevant literature and editing the manuscript. All authors gave approval of the final version submitted.

### Conflict of interest statement

The authors declare that the research was conducted in the absence of any commercial or financial relationships that could be construed as a potential conflict of interest.
